# A comparison of two systems of patient immobilization for prostate radiotherapy

**DOI:** 10.1186/1748-717X-9-29

**Published:** 2014-01-22

**Authors:** Peter White, Chui Ka Yee, Lee Chi Shan, Lee Wai Chung, Ng Ho Man, Yik Shing Cheung

**Affiliations:** 1 The Hong Kong Polytechnic University, Hung Hom, Kowloon, Hong Kong

**Keywords:** Immobilization, Hipfix, Alpha cradle, Set-up errors, Treatment verification, CTV-PTV margins, Prostate cancer

## Abstract

**Background:**

Reproducibility of different immobilization systems, which may affect set-up errors, remains uncertain. Immobilization systems and their corresponding set-up errors influence the clinical target volume to planning target volume (CTV-PTV) margins and thus may result in undesirable treatment outcomes. This study compared the reproducibility of patient positioning with Hipfix system and whole body alpha cradle with respect to localized prostate cancer and investigated the existing CTV-PTV margins in the clinical oncology departments of two hospitals.

**Methods:**

Forty sets of data of patients with localized T1-T3 prostate cancer were randomly selected from two regional hospitals, with 20 patients immobilized by a whole-body alpha cradle system and 20 by a thermoplastic Hipfix system. Seven sets of the anterior-posterior (AP), cranial-caudal (CC) and medial-lateral (ML) deviations were collected from each patient. The reproducibility of patient positioning within the two hospitals was compared using a total vector error (TVE) parameter. In addition, CTV-PTV margins were computed using van Herk’s formula. The resulting values were compared to the current CTV-PTV margins in both hospitals.

**Results:**

The TVE values were 5.1 and 2.8 mm for the Hipfix and the whole-body alpha cradle systems respectively. TVE associated with the whole-body alpha cradle system was found to be significantly less than the Hipfix system (p < 0.05). The CC axis in the Hipfix system attained the highest frequency of large (23.6%) and serious (7.9%) set-up errors. The calculated CTV to PTV margin was 8.3, 1.9 and 2.3 mm for the Hipfix system, and 2.1, 3.4 and 1.8 mm for the whole body alpha cradle in CC, ML and AP axes respectively. All but one (CC axis using Hipfix) margin calculated did not exceed the corresponding hospital protocol. The whole body alpha cradle system was found to be significantly better than the Hipfix system in terms of reproducibility (p < 0.05), especially in the CC axis.

**Conclusions:**

The whole body alpha cradle system was more reproducible than the Hipfix system. In particular, the difference in CC axis contributed most to the results and the current CC margin for the Hipfix system might be considered as inadequate.

## Background

### Incidence

Prostate carcinoma is one of the most prevailing malignant diseases in men [1]. Recently, findings have indicated that prostate cancer, which accounted for 10.7% of all cancers in 2010, is ranked third among all male cancers in Hong Kong [2]. However, compared with other malignancies, prostate tumours are comparatively of torpid progress and are less prone to metastasize. Thus, they are less prone to cause significant morbidity and mortality [3]. With advancements in diagnostic technology, such as the measurement of serum prostate-specific antigen (PSA), early stage, localized prostate cancer is usually detected [4]. Hence, according to the National Cancer Institute [5], 81% of prostate cancer patients present with localized disease. As a result, because of its high incidence and low mortality rate, localized prostate cancer is worth investigating and its management is of paramount significance.

### Radiotherapy techniques

Along with radical prostatectomy, one of the two major standard curative treatments, external beam radiotherapy (EBRT) is a more prevalent curative treatment modality for localized prostate cancer [6]. With similar biochemical relapse-free survival rates [7], EBRT shows better performance in minimising side-effects such as incontinence and impotence [8,9]. For prostate EBRT, rectum, small bowel, bladder, bilateral femoral heads and penile bulb are incorporated in organs-at-risk (OARs) [9]. Among the sophisticated EBRT techniques, including intensity-modulated radiotherapy (IMRT), volumetric-modulated arc therapy (VMAT) is often used in treating localized prostate cancer due to its relatively short treatment delivery time, higher dose conformity and homogeneity [10,11], as well as better OARs sparing [6]. VMAT is a special type of dynamic IMRT which allows four parameters to modulate simultaneously, which include gantry rotation speed, output rate (dose rate), multi-leaf collimator (MLC) orientation and leaf speed [12-14]. In Hong Kong, the current commercially available VMAT is RapidArc (RA) by Varian Medical Systems and, as such, VMAT and RapidArc are used interchangeably in this study.

Effective utilization of VMAT technique relies on highly accurate radiation delivery [15] and inaccurate positioning may result in under-dosage of the treatment target volume [16]. Therefore, as the significance of immobilization to set-up reproducibility, which is determined by the shift of isocentre [17], has been well documented and ascertained in numerous studies [17-22], a rigid and reliable immobilization system is required [23] to enhance the efficacy of VMAT.

### Immobilization device accuracy

One study previously compared the set-up reproducibility between a conventional treatment position (CTP) and an immobilization system (IMS) [24]. CTP provided immobilization of patients through a foam head pad and standard ankle stocks, while IMS allowed patients to lie on a customized cushion supporting them from iliac crests to upper thighs. Despite the minor increment of patient comfort using IMS, the study did not prove any enhancement in treatment accuracy.

Another study compared the effectiveness of immobilization of the legs with alpha-cradle and that of the pelvis and abdomen with alpha-cradle in terms of a retrievable set-up [23]. Results manifested improved reproducibility of positioning of patients using alpha-cradle leg immobilization over the latter. However, a similar study [16], which compared the set-up reliability of: 1) a leg cushion, 2) a whole body alpha cradle, and 3) a thermoplastic Hipfix system, had completely contrasting results. It was concluded that the differences in reproducibility of the three patient immobilization devices were statistically significant. In particular, the Hipfix, which was an immobilization system surrounding the abdominal and pelvic region, performed best in terms of set-up error reduction.

Song et al. [25] further refined these results. Using no immobilization versus four immobilization systems, including: 1) alpha-cradle from waist to upper thigh, 2) alpha-cradle from hip to knee, 3) a leg cushion, and 4) a thermoplastic cast surrounding the entire abdomen and pelvis to the mid-thigh with alpha-cradle immobilization of lower legs and feet, comparisons were made in terms of the variability in positioning obese patients throughout the radiotherapy course. No significant reduction in overall patient movement by any of the four immobilization systems was shown, although the thermoplastic cast immobilization system performed better in two directions (anterior-posterior and cranial-caudal). Alarmingly, the study emphasized a lack of optimal immobilization and patient positioning systems in treating prostate cancer with EBRT.

Therefore, comparisons between the effectiveness of various immobilization devices remain inadequate and inconsistent [16], at least for supine patients [23], although research has demonstrated greater improvement in set-up reproducibility from none at all to a certain degree of immobilization [16]. In addition, there is inconsistency in terms of the immobilization systems used in Hong Kong and the patient size and shape tends to differ from foreign countries. Therefore, a study of the set-up reproducibility between different immobilization devices is valuable so as to improve the VMAT potency and, hence, benefit patients within the local setting [19].

### Adverse effects due to set-up errors

The role of immobilization as an essential tool to compensate the occurrence of set-up errors has been documented [23]. Set-up errors, which are inevitable within the radiotherapy course, increase the chance of inadequate dose to the target as well as unnecessary irradiation of adjacent normal tissues [26]. For patients with prostate cancer, the increase in set-up errors will eventually lead to higher local relapse and more severe radiation reactions, such as diarrhea and radiation cystitis [6]. This geometric uncertainty necessitates the employment of larger field margins (margins between the target volume and the field edges) in order to achieve homogeneous irradiation of the target volume [27].

### Other factors contributing to geometric uncertainties

Apart from set-up variations, organ motion and target volume delineation also contribute to geometric uncertainties [28,29]. The daily variation of prostate location can be minimized by consistent rectal evacuation and bladder preparation before treatment [30]. Also, clips surgically inserted into the prostate gland (fiducial markers) can demonstrate prostate motion through improved treatment verification [31]. Stable bony landmarks are often employed to aid the determination of soft tissue motion [30]. Nevertheless, target volume delineation is influenced by inter-observer variability. Even though the situation can be improved through well-defined treatment protocols, as well as experienced oncologists, the inevitable existence of some uncertainties has been emphasized [30].

### Margin delineation

According to ICRU report 50 [32] and its refinement in IRCU report 62 [33], PTV has been introduced to determine field directions, shapes and weightings and thus is responsible for the dosimetric calculations and presentations. PTV is delineated from the gross tumour volume (GTV) in two steps. GTV is first enlarged into CTV to include the subclinical microscopic spread of disease. CTV is then further expanded to PTV with regard to the organ motion and set-up errors [27,32,33]. In other words, PTV is a direct function of set-up margins [26]. In essence, the CTV-PTV margin is determined with respect to geometric uncertainties.

### Aims and objectives

The aims of this study were to improve the potency of VMAT by achieving more precise patient positioning and to study the corresponding CTV-PTV margins. The ultimate goal was to benefit both staff and patients directly through easier reproducibility of the treatment position as well as reducing treatment times, improving local control and reducing radiation side-effects respectively. With reference to RapidArc treatment for prostate cancer, the objectives of this project were to:

1. Compare the set-up reproducibility of patient positioning with Hipfix and alpha cradle for T1-T3 prostate cancer patients.

2. Investigate the existing CTV-PTV margins in the clinical oncology departments of two regional hospitals.

## Methods

### Patient recruitment and selection

This was a retrospective study conducted in the clinical oncology departments of two regional hospitals in Hong Kong. Ethics approval was granted for this study by the Departmental Research Committee, on behalf of the Human Subjects Ethics Sub-committee, at The Hong Kong Polytechnic University. Forty prostate cancer patients, 20 from each hospital, were randomly recruited. Stage T1-T3 prostate cancer cases, with no distant metastases, were selected. The sample size was statistically generated by power analysis using the latest G*Power 3.1.5 software with α = 5%, 1 - β = 80% and effect size ≈ 0.9585, as estimated from a similar study [16]. The staging of patients was determined as localized disease encompasses the majority of cases in prostate cancer [4].

### Data collection

**Figure 1:**
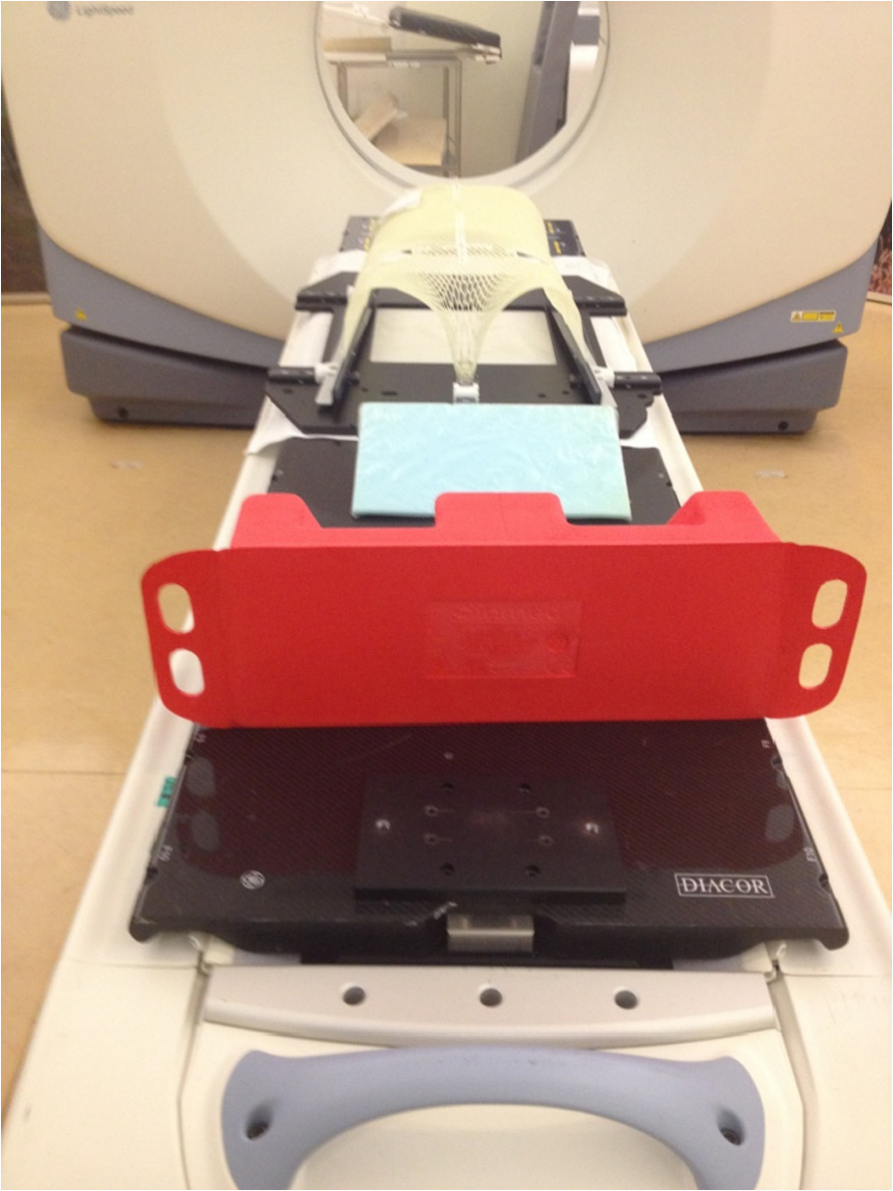
Illustration of immobilization system in Hospital A.

**Figure 2:**
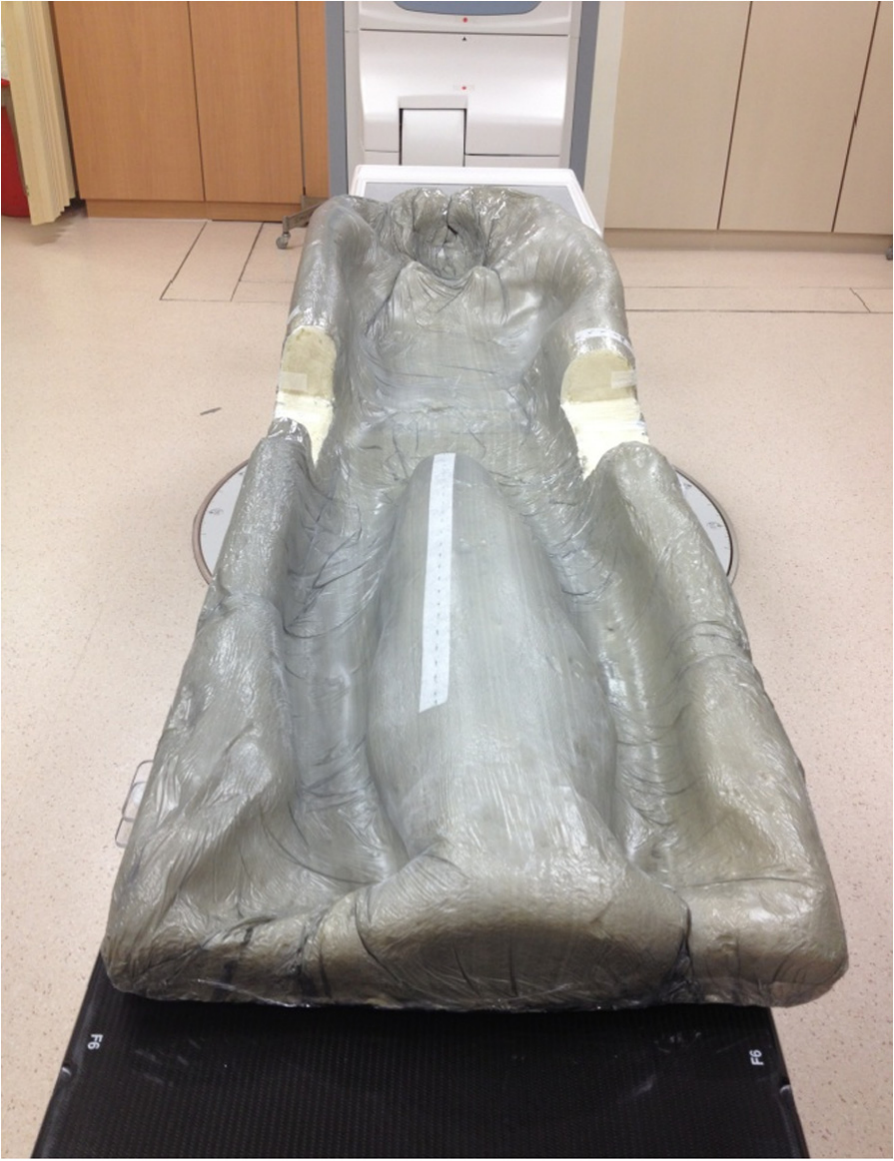
Illustration of immobilization system in Hospital B.

Currently, various immobilization systems are employed to treat prostate cancer by EBRT in Hong Kong. The major two are: 1) a thermoplastic Hipfix system from waist to upper thigh with a Feetfix (Figure 1) and 2) a whole body alpha-cradle (Figure 2). These systems are described as using Hipfix and alpha-cradle respectively in the following content. Hipfix was employed by Hospital A, while alpha-cradle was adopted by Hospital B. The study was supported by both hospitals in terms of patients’ data collection, which included the isocentre shifts of the patients (the x, y and z dimensions). Apart from the fact that they employed the major immobilization devices, both hospitals were selected because of their highly comparable protocols for localized prostate cancer EBRT with VMAT (Table 1).

**Table 1:** Comparison between protocols in Hospitals A and B

	**Hospital A**	**Hospital B**
Treatment technique	RapidArc	RapidArc
Immobilization devices	Hipfix	Alpha-cradle
Dose scheme	2 Gy at 100% isodose level per fraction, 5 fractions per week, to a total dose of 76 Gy in 38 fractions	
Bladder preparation	Drink 400 ml of water 60 minutes prior to the scan or treatment	
Rectal evacuation	N.A.	Bisacodyl 10 mg (oral) before bedtime is prescribed to empty bowel before the night of planning CT and each treatment unless the patient was suffering from diarrhea

It is noted that Hospital A did not take measures for rectal evacuation. Nevertheless, literature states that an empty rectum protocol generally does not reduce the variability of prostate position [34]. Therefore, bias in favour of the alpha cradle system in Hospital B could be ruled out.

### Immobilization and positioning

In both hospitals, patients lay in supine position with their legs slightly abducted. The Hipfix system in Hospital A included a ready-made Hipfix baseplate with a cut-out treatment window, a Styrofoam Feetfix and a tailor-made auqaplast sheet. The aquaplast sheet, which was a synthetic polymer [25], was heated to 77-81°C in a water bath. Due to its malleability after heating [16], the pliable sheet was then stretched and moulded to conform to the patient’s external contours from waist to upper thigh. The sheet was locked into the baseplate on two lateral sides and between patients’ thighs. It was then allowed to cool down and harden. The baseplate was not indexed to the couch. The Feetfix was used to assist in securing the lower legs, while a foam pad was used to ensure patient comfort.

In Hospital B, the immobilization system consisted of a metallic bar, a baseplate and an individually customized alpha-cradle with a cut-out chest support. First, a baseplate was locked onto the couch by an indexed metallic bar. The alpha cradle was chemically formed using two foaming agents [16,25]. After mixing and placing into a thick plastic bag with a cut-off chest support inside, the two agents reacted and expanded around the patient’s body. With the slightly abducted position, the patient’s legs were also supported by alpha cradle in between the legs. After the hardening procedure, a stable, solid repositioning device was formed [16,25].

### Computed Tomography (CT) simulation

Before CT simulation and each treatment, the patient was instructed to empty his bladder and then drink water accordingly. Hospital A adopted a scheme whereby 400 ml of water should be drunk 60 minutes prior to the scan or treatment, while in Hospital B, patients were asked to drink 400 ml of water 60 minutes before both procedures. For Hospital B, with regard to bowel preparation, the patient was prescribed with bisacodyl 10 mg (oral) before bedtime to empty the bowel before the night of planning CT and each treatment, unless the patient was suffering from diarrhea, while no bowel preparation was required for routine prostate cases in Hospital A.

The treatment planning CT scan was performed with 3-mm slices throughout. Coverage of the scan was from iliac crest to perineum in Hospital A and from the third lumbar vertebra to anus (about 5 cm below ischial tuberosity) in Hospital B. Patients were positioned on customized immobilization devices. The preliminary principal plane (PP), horizontal level (HL) and midline (ML) were obtained with the aid of a laser system, which was under regular surveillance. Contrast medium was used to outline pelvic lymphatics. In Hospital A, PP and HL were drawn on the Hipfix only, while ML was drawn on both skin and the aquaplast. For Hospital B, cutaneous landmarks consisted of PP and ML, while all three directions were indicated on the alpha-cradle. Digitally reconstructed radiographs (DRRs) were then prepared after treatment planning for setup verification.

### Radiotherapy planning technique

The GTV, seminal vesicles and OARs, were determined and delineated on the CT images by oncologists with regards to protocols. The CTV and PTV were then generated by prescribing margins to the GTV. The target volume protocols of both hospitals are indicated in Table 2.

**Table 2:** Comparison of the target volume protocols for Hospital A & B

	**Hospital A**	**Hospital B**
GTV	Prostate	N.A.
CTV	Prostate + proximal bilateral seminal vesicle (SV)	Prostate (+ whole SV if involved)
PTV	CTV + 1 cm (0.5 cm for posterior margin)	CTV + 0.5 to 1 cm (1 cm margin is usually used except 0.6 cm posteriorly)

It can be observed that both protocols were similar. The GTV or CTV was contoured by experienced oncologists. After the determination of target volumes and normal tissues, RapidArc treatments using inverse planning were completed by experienced radiation therapists and oncologists. All forty patients were treated with 2 Gy at 100% isodose level per fraction, 5 fractions per week, to a total dose of 76 Gy in 38 fractions.

### Treatment verification

The verification procedures were firstly done in the simulator unit. Patients were positioned using the same set-up as for the previous CT-simulation. Relatively stable bony structures, which included sacro-iliac joints, lumbar vertebrae, iliac crests, obturator foramen, pubic bone and ischial tuberosities, were used for matching the current position with DRRs. Orthogonal check films were obtained during fluoroscopy. PP and HL marks were shifted to the level of the planning isocentres.

The remaining series of verifications were performed in the treatment units. Before the treatment course, patients were positioned using a three-point set-up technique [25], with the aid of optical field crosswires, a laser system and an optical distance meter. The Varian Medical Systems On-board imager (OBI) was manipulated to obtain orthogonal images. Similar to the verification steps conducted in the simulator, each patient’s position was finely adjusted to the designated treatment position by matching the OBI images with DRRs with respect to bony landmarks.

This included four directions: couch angle , medial-lateral (ML), cranial-caudal (CC) and anterior-posterior (AP). The deviations, which could be expressed in terms of angular and translational (x, y, z) displacements, were measured and shown on the OBI console. All positioning and matching procedures were carried out by qualified radiation therapists so as to minimize any threat to internal validity. Since the OBI system manifested the actual dimensions of deviations, there were no magnification problems in the superimposing and matching process.

Daily and weekly OBI was performed by Hospitals A and B respectively. Therefore, it was appropriate to collect seven sets of displacements for each patient with each set of data collected on the first day of treatment each week.

### Reproducibility estimation

Total vector error (TVE) was employed to determine overall shift from the simulation isocentre. TVE is a mathematical function which takes ML, CC and AP errors, which are represented by x, y and z respectively, into account simultaneously, where *TVE* = (*x*^2^ + *y*^2^ + *z*^2^)^1/2^[16]. As angular displacement does not affect the isocentre matching, it was not included in this estimation. A smaller TVE indicates higher reproducibility of the immobilization device, so this calculation formula was adopted as it is used worldwide [16,28,35].

In essence, the mean of 7 TVE values within a patient was computed. As a result, twenty means of TVE from each hospital were compared.

### Determination of optimal PTV margins

As the target delineation procedures were overseen by experienced oncologists using well-defined protocols, the variation of target volume contouring was minimized. Meanwhile, the organ motion was estimated by bony landmarks and bladder preparation. Therefore, the CTV-PTV margin determination in this study was based on the isocentre shifts.

Van Herk [28,35] suggested a formula to estimate CTV-PTV margins by ML, CC and AP errors indirectly. PTV margin = 2.5Σ + 0.7σ, where Σ and σ are the systematic and random errors respectively. The ML, AP and CC errors were manipulated separately to estimate their own margins in the corresponding direction. This formula was adopted as it is widely used and has been justified in previous studies.

The ML margin is employed as an example. On the one hand, the systematic error in ML direction is estimated as follows. As 7 values of x were obtained per patient, a total of 20 values of mean of x within each patient $\left({\bar{x}}_{1},{\bar{x}}_{2},{\bar{x}}_{3}\ldots {\bar{x}}_{20}\right)$ were generated. The systematic error in ML direction was defined as the standard deviation (SD) of $\left({\bar{x}}_{1},{\bar{x}}_{2},{\bar{x}}_{3}\ldots {\bar{x}}_{20}\right)$[26,30,36]. On the other hand, random error was computed by calculating SD within a patient by using the 7 values of x followed by the root mean square of SD. In other words, random error was defined as the root mean square of SD of all patients [26,30,36]. The systematic and random errors of the CC and AP directions were determined in the same way.

### Statistical analysis

Data analysis was conducted using both the Statistical Package for Social Sciences (SPSS) version 9.0 and Microsoft Office Excel 2010. The level of significance (α) was set to 0.05.

First, the normality of the data was tested by Shapiro-Wilkes test. If they were normally distributed (p > 0.05), the equality of variances of two groups were tested by Levene’s test. Equal variances were assumed when p > 0.05, while p < 0.05 indicated an assumption of unequal variances. As the two groups of data were independent, two-tailed independent samples *t*-test were performed. Otherwise, if they were not normally distributed (p < 0.05 in Shapiro-Wilkes test), a non-parametric test, two-tailed Mann–Whitney *U* test, was employed.

The null hypothesis was *μ*_1_ = *μ*_2_ whereas the alternate hypothesis was *μ*_1_ ≠ *μ*_2_, where *μ*_1_ and *μ*_2_ were the population mean of TVE using two immobilization systems. A p-value <0.05 indicated the rejection of the null hypothesis, which meant that the difference between two groups was significantly different.

## Results

In total, 140 measurements of set-up errors in each direction were collected from the OBI console, so that 7 sets of measurements were obtained per patient.

It was observed that the systematic shifts in set-up errors for each patient would lead to non-zero overall deviations in either direction. Nevertheless, literature has shown that the set-up errors follow a normal distribution around the isocentre [37,38]. This concurred with the findings as both sets of data, from Hospitals A and B, were normally distributed (p = 0.193 and p = 0.054 respectively).

### TVE and mean of absolute differences

It was not reasonable to assume systematic set-up deviations in any specific direction [16]. As a result, instead of considering the direction within an axis, the mean of absolute differences for each axis and for TVE were calculated. Moreover, the standard deviations were manipulated to show the variability of the corresponding mean of absolute differences. Detailed comparisons are summarized in Table 3.

**Table 3:** Mean absolute differences for each axis and TVE

	**Mean of absolute differences (mm) for each axis and for TVE**			
	**AP**	**CC**	**ML**	**TVE**
Alpha cradle	0.8 ± 0.4	1.3 ± 0.5	1.8 ± 0.9	2.8 ± 0.8
Hipfix	1.0 ± 0.6	4.3 ± 2.1	1.3 ± 0.4	5.1 ± 1.9
	p = 0.091	p = 0.000	p = 0.051	p = 0.000

(Data presented as mean ± standard deviation).

The differences in CC axis and TVE value were statistically significant (p = 0.000). The mean of absolute difference in CC axis for Hipfix was greater than for alpha cradle (p = 0.000). Also, the TVE value for alpha cradle was significantly less than for Hipfix. Though it was not statistically significant (p = 0.051), the mean of absolute difference in ML axis for alpha cradle was greater than for Hipfix.

### Incidence of set-up errors greater than 5 mm or 10 mm

**Figure 3:**
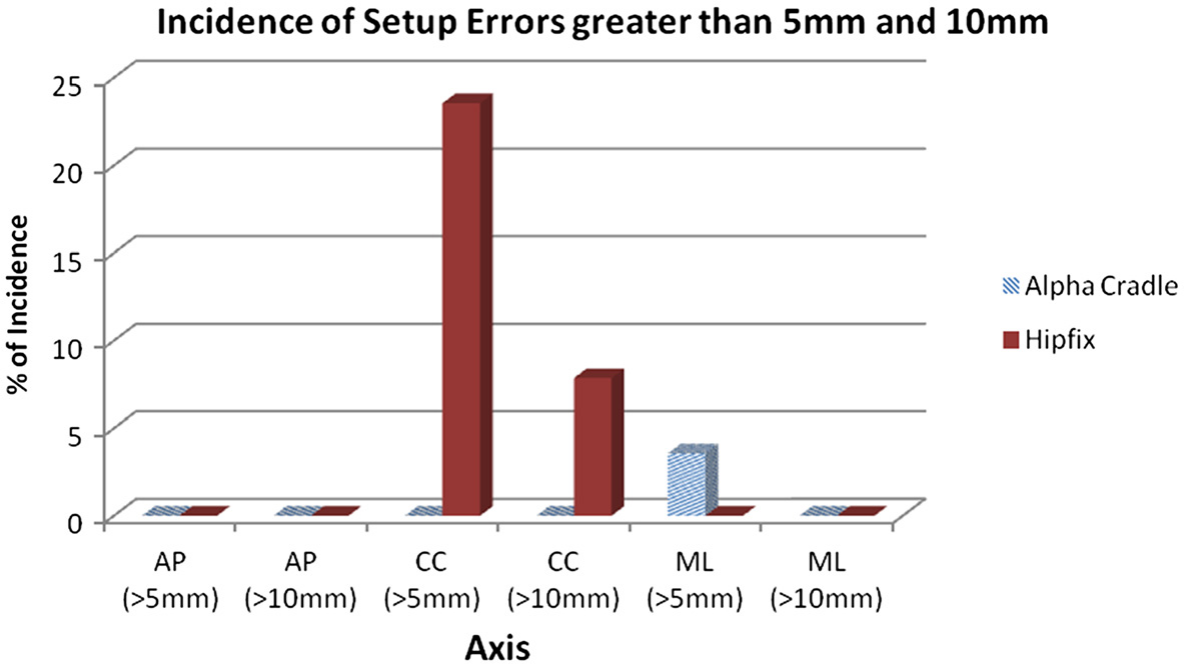
Incidence of set-up errors greater than 5 mm and 10 mm for all three axes.

The incidences of set-up errors greater than 5 mm or 10 mm are illustrated in Figure 3. For set-up errors greater than 5 mm, the CC axis for Hipfix system had the highest incidence, that is, 23.6%, while the ML axis for alpha cradle system was only 3.6%. For set-up errors greater than 10 mm, the CC axis for Hipfix system showed the highest incidence, which was 7.9%. For the remaining fixed axes, set-up errors greater than 10 mm were virtually eliminated.

### Margins calculated

The margins calculated for each axis using the two immobilization systems are shown in Table 4. For the alpha cradle system, the margin calculated in ML axis was the largest, while the AP axis was the smallest. By contrast, for the Hipfix system, the margin calculated in ML axis was the smallest. The CC axis had the largest margin for Hipfix system.

**Table 4:** Systematic errors, random errors and margins calculated for each axis (in mm)

		**Systematic error**	**Random error**	**Margin calculated**
AP	Alpha cradle	0.42	1.0	1.8
	Hipfix	0.58	1.2	2.3
CC	Alpha cradle	0.48	1.2	2.1
	Hipfix	2.13	4.2	8.3
ML	Alpha cradle	0.92	1.5	3.4
	Hipfix	0.44	1.1	1.9

## Discussion

It has been widely observed that immobilization devices are important in positioning reproducibility [21-25,28]. In this study, the relative impact of HipFix and alpha-cradle on the reproducibility during prostate radiotherapy was evaluated.

### Higher reproducibility for alpha cradle system

With a lower TVE value (Table 3), the alpha cradle had a smaller overall isocentre shift than the Hipfix system, so was more reproducible, which contradicted a previous study [16]. However, both studies were not fully comparable as the alpha cradle used in the previous study covered only from mid-thorax to below the feet. By contrast, the alpha cradle used in our study covered from head to feet. The deviation in results may be due to the fact that the larger the coverage and extension of the immobilization system, the higher the reproducibility, though other literature has indicated there may be a saturation value for this phenomenon [39]. Another difference between these studies which may cause deviations in the results is the patient position. In our study, Hospital A employed Hipfix system to position the patient in a supine manner, while in the other study the patients were prone [16].

Research has shown rigid leg immobilization systems to be more reproducible than rigid pelvic-abdominal ones [23]. This was found when considering the average isocentre shifts, as improvements when using leg immobilization compared with alpha cradle at pelvis level were considerably higher. Thus, comfortable leg position is of paramount importance in the set-up of supine patients [23]. The alpha cradle system, which was tailor-made for the entire lower limb region, proffered greater reproducibility.

The materials used for constructing immobilization systems may have also contributed to the results. Some studies have stated that alpha cradle is less elastic or flexible than a thermoplastic cast [39,40]. In other words, alpha cradle is more rigid and is less likely to shrink or change shape. These features may have attributed to the results since an unexpected change in the immobilization system adversely influences its reproducibility. As a result, the reproducibility of Hipfix system appeared to be inferior to the alpha cradle system.

In addition, the frequency of verification imaging may have also influenced the results. As Hospital A performed daily verification using OBI for routine localized prostate cancer patients, the staff may be more reliant on these images. As they performed verification before each treatment fraction, they may consider the images to be the final reference when positioning patients. This made them less aware of the finer accuracies between the skin marks, as well as tattoos and the laser beams, while positioning the patients.

### Mean absolute differences

The differences in mean absolute differences of CC axis were statistically significant, while those of AP and ML axes were not (Table 3).

In the AP direction, the performance of both immobilization devices was similar, contradicting previous studies [16,23,24]. This may be due to the fact that both immobilization systems were comfortable for patients, as it has been stated that comfortable leg immobilization reduces patient rotation. This kind of rotation may attribute to shifts of the skin marks and tattoos on the lateral aspects of the patients, and eventually cause apparent AP shifts [23].

For the CC axis, alpha cradle system was significantly better than the Hipfix system, but the latter performed better in ML direction. Both findings agreed with another study, which showed that the physical design of the thermoplastic system is rigid in ML axis, but relatively less rigid in CC axis [16]. Moreover, as the alpha cradle system covered the whole body, the motion in CC direction of patients within the alpha cradle was very limited.

### Incidence of set-up errors greater than 5 mm or 10 mm

The usage of 5 mm or 10 mm set-up errors has been justified in previous studies [16,23]. The CC direction using Hipfix system accounted for the highest incidence of large set-up errors (Figure 3). In the literature [16,23], large and serious set-up errors, which are errors greater than 5 mm and 10 mm respectively, were most commonly found in CC direction too. This is attributed to the tendency for rotation of hips, which predominantly affects the set-up errors in CC axis. As a result, this directional predominance of set-up deviations for pelvic irradiation was supported by current findings. There were also occasions of large set-up errors occurring in ML direction using alpha cradle. This was probably due to weight loss of patients [16], as alpha cradle does not perform as well in ML direction under such circumstances [25].

Set-up errors greater than 10 mm were virtually eliminated in all axes except the CC axes using the Hipfix system (Figure 3), so both immobilization systems performed satisfactorily in avoiding serious set-up errors.

### Systematic and random errors

A systematic error is defined as the variation of the mean displacement of patients [40]. It can also be regarded as the difference between the planned patient position and the average patient position throughout a fractionated course of radiation therapy. Random error is defined as day-to-day variation during a treatment series [40].

From Table 4, random errors were generally larger than systematic errors. One study also investigated the systematic and random errors for pelvic irradiation [23]. In particular, for patients with prostate cancer, random errors were found to be larger than the systematic errors [23]. However, another study concluded that the systematic component was larger than the random one. This contradiction in findings may be due to the advancement of techniques and technology. In fact, systematic error is much more likely to be reduced or eliminated than random error, as the latter is intrinsic and is very difficult to correct [40]. With improvements in radiotherapy, the ratio of systematic errors to random errors would decrease gradually. It is also reasonable to find that the systematic errors calculated in this study were generally smaller than those previously [23], while random errors of both studies were comparable. In the light of this phenomenon, further investigations are required to further reduce the systematic component so as to minimize errors.

Both immobilization systems offered comparable results for systematic and random errors in the AP axis. In CC axis, alpha cradle showed a substantial reduction in both systematic and random errors, while in ML axis, the Hipfix system provided slightly smaller systematic and random errors. Therefore, following on from the previous discussion about TVE values and means of absolute differences, it can be concluded that alpha cradle performed better in reducing these two types of error.

### Margins calculated

As shown in Table 4, it was found that the axes for largest and smallest calculated margins for both immobilization systems were different. This pattern difference was probably due to the physical design of the immobilization systems, as thermoplastic Hipfix system was found to be rigid in ML direction, but relatively less rigid in CC axis [21]. Moreover, the rigidity of alpha cradle minimized any change in its shape due to the patient’s weight in AP axis. As there was no anterior cover to the patient’s skin anteriorly, rotation of the patient was likely to be seen using the alpha cradle system [24]. This was probably the reason for the largest margin being calculated in ML direction for the alpha cradle system. Hence, ideally, for each immobilization system, there should be a particular margin for each direction. Actually, both Hospitals A and B had implemented this notion into their posterior margin as it was usually smaller than margins in other directions. However, this reduction in margin is mainly due to the consideration of rectal dose, but not set-up errors. This is of clinical significance as different margins should be used for each axis.

The second point is that no margins exceeded those in the current protocols (Table 5). Nevertheless, the margins calculated only accounted for set-up errors, but not organ motion and target delineation.

**Table 5:** Comparison between protocols of hospitals and margins calculated

**Immobilization device**	**Protocol of hospitals**	**Margins calculated for each axis (mm)**		
		**AP**	**CC**	**ML**
Whole body alpha-cradle	0.5 to 1 cm (1 cm margin is usually used except 0.6 cm posteriorly)	1.8	2.1	3.4
Hipfix system	1 cm (0.5 cm for posterior margin)	2.3	8.3	1.9

For the sake of comparison, the overall CTV-PTV margins for each case were estimated. Previous studies have suggested that the dimensions of prostate movement are above or below 5 mm, with predominance in AP and CC axes [16,41]. Furthermore, literature has reported that the means of prostate motions in AP, CC and ML axes were 6 mm, 5.9 mm and 0.5 mm respectively [42]. Since these movements must be considered when designing the PTV [16], these dimensions were added into calculated margins so as to more accurately estimate margins and more reliably compare them with the ones in hospital protocols. The detailed comparison between protocols of hospitals and margins calculated after accounting for prostate motion in each axis, which are called modified margins in the following content, is illustrated in Table 6.

**Table 6:** Comparison between protocols of hospitals and modified margins

**Immobilization device**	**Protocol of hospitals**	**Modified margins for each axis (mm)**		
		**AP**	**CC**	**ML**
Whole body alpha-cradle	0.5 to 1 cm (1 cm margin is usually used except 0.6 cm posteriorly)	7.8	8	3.9
Thermoplastic Hipfix system	1 cm (0.5 cm for posterior margin)	8.3	14.2	2.4

Due to the rectum, the posterior margins were expected to exceed corresponding protocol requirements. This means that with regard to the posterior direction, insufficient radiation dose would be delivered. This is somehow inevitable so as to prevent severe and unacceptable toxicity of the rectum. This situation may result in poor local control and higher risk of local relapse [43].

However, a further observation is that, after the addition of corresponding prostate motion, the modified margin for Hipfix system in CC direction exceeded 1 cm. This would appear to suggest that the current margin in CC direction for Hipfix system in Hospital A was inadequate. However, as daily OBI was performed in Hospital A to rectify the set-up errors, then these concerns are unfounded. It can also be observed that the modified margins in ML axis for both immobilization systems were significantly smaller than their corresponding protocols. It could be suggested that the current CTV-PTV margins for the left and right directions have the potential to decrease. The reduction of margin allows not only dose escalation but also reduction of unnecessary irradiation of adjacent normal tissues [26].

#### Uncertainties about skin marks

As the set-up process in both Hospitals A and B relied on the skin tattoos and skin marks, their reliability played a crucial role in reproducibility. However, it is common for the radiation therapists to find that the set-up marks on the patient’s skin ‘migrate’. This occurrence may be due to the ‘touching up’ of the skin marks by radiation therapists on a day to day basis [21]. As the skin marks gradually fade away, if they are consistently replaced a bit to one side, then, after a period of time, the skin marks could be displaced by a significant distance. The fading and blurring of skin marks also affects the accuracy of patient positioning. Unfortunately, these are difficult to detect, correct or quantify. To minimize the adverse effects due to skin mark migration, one should draw extra set-up marks on immobilization systems [21]. In both hospitals, initial skin marks may be adjusted after consecutively large OBI deviations. As a result, the deviations obtained afterwards may be affected. Nevertheless, this threat to internal validity could be minimized by experienced and qualified radiation therapists as well as random sampling.

#### Retrospective basis

As our study was retrospective, it was restrictive in that patient-related information, such as the dimensions of change in patient size during the course of treatment, could not be obtained. Literature has shown that patient size affects the reproducibility of an immobilization system [25,41,43]. The size and shape of the patients, including parameters such as pelvic circumference, are examples of the most commonly investigated factors. Song et al. [25] reported that obese patients and/or patients with pelvic circumferences greater than 105 cm attributed to diminished reproducibility, especially in ML axis. Hurkmans et al. [36] suggested that daily online imaging and positioning corrections were valuable for obese patients. Even if the pelvic circumferences are the same, the shape of patients can vary. Some patients may have a more circular body outline, while some may have a more elliptical one. This relates to the separations in AP and ML axes. Moreover, change of size, shape and circumference of the patient during the radiotherapy course affects reproducibility [21]. As a result, the scope of this study was limited by the retrospective design, and further prospective investigations are recommended.

#### Use of bony landmarks

With reference to the verification images obtained, deviations in position of bony landmarks were used to estimate and represent the position of the target volume and prostate. Literature has indicated that bones are not good surrogates for the prostate as it is an organ which regularly changes shape and volume and experiences internal movement [43]. In order to deliver the radiation treatment more accurately, more sophisticated verification techniques such as cone-beam CT and the Calypso real-time tracking system are recommended [44]. Implantation of fiducial markers into the prostate is another alternative to better represent the location of the target volume [40].

#### Influence on reproducibility by mobility of patients

The mobility of individual patients is one of the extraneous variables. Besides involuntary skin movement due to respiration or variable filling of bladder and rectum [36,45], mobility depends on age, staging, physical state and compliance of the patients. A positive relationship between T-stage and the dimension of set-up errors has previously been reported [46]. It was observed that patients with more symptomatic advanced prostate carcinomas found it more difficult to maintain a full bladder state, and hence it may be more difficult for them to maintain a stable position within the immobilization system. Also, elderly patients often have flaccid skin which makes the position of skin tattoos or skin marks relative to the target volumes, uncertain [21]. The physical state refers not only to any concurrent diseases such as asthma but also radiation reactions including skin reactions and perianal discomfort, which are often encountered by prostate patients [21]. Apart from physical state, mental state is influential too [36]. Therefore, the final mobility-related factor is the compliance of patients. Some patients may not be compliant or some may contract their muscles due to anxiety and, thus, they may be unstable and are more likely to cause large or even serious set-up errors.

### Clinical value

As the majority of routine localized prostate cancers in Hong Kong employ Hipfix or alpha cradle systems with OBI verification, the following clinical implications should be noted.

First, each immobilization device has its own strengths, such as reducing set-up errors in a particular axis. After determining the strengths and weaknesses, modifications or even combinations of the systems could be used so as to obtain improved immobilization.

Second, customized margins for each axis, or even each direction, were essential to tailor-make the PTV. Customized margins should be considered in the future.

### Recommendations and further research

Firstly, in order to reduce set-up errors in the CC direction, it has been suggested to place tattoo marks on the lateral aspects of the patient at isocentre level so as to line up with the zero mark on the immobilization system [16].

Secondly, to improve reproducibility by immobilizing the whole patient, Bentel et al. [21] extended their casts further caudally to include the feet. Extra skin marks and set-up marks could then be placed on the patient’s tibia and adjacent cast respectively. Compared with only focusing on the treatment region, these actions could confirm the proper positioning of the patient within the whole immobilization system. This should also be balanced and justified with the consideration of resources.

Thirdly, although there was no statistical significance in the means of absolute difference in AP direction of both immobilization systems, the accuracy in this direction is of paramount importance in treating localized prostate cancers. This is due to the anatomical structures as two major OARs in prostate radiotherapy, bladder and rectum, are located adjacent to the anterior and posterior edges of the prostate [45].

An additional set-up parameter called isocentre-couch distance has been shown to introduce a significant reduction of set-up errors in AP axis [36]. This idea has already been adopted in some oncology departments in Hong Kong, but its wider use is recommended.

Fourthly, Perera et al. [47] found that it was very difficult for human observers to accurately identify set-up errors less than 5 mm when using manual methods. Therefore, the employment of automatic registration should be considered whenever possible. As a result, further investigation is required. Prospective design of the research is highly recommended so as to include the maximum amount of information in the interested area.

Since current patient setup procedures are highly reliant on the alignment of external skin marks, this causes extraneous variables in the reproducibility of immobilization systems. On the one hand, skin marks contain a certain level of uncertainty which includes ‘touching up’ and blurring [16]. On the other hand, there is no consistency in the position of skin marks relative to the target volume [45]. Therefore, more direct methods, such as patient positioning and set-up by the aid of internal markers, could be included.

Finally, 3D or 4D verifications better reflect any deviation of target volumes between the period of treatment and planning. Similar or more in-depth research with cone-beam CT, room mounted kV fluoroscopic imaging, etcetera is anticipated. Besides, the investigation of more than three axes would be expected in the future. This could be done via 6D couch to account for more precise patient positioning.

## Conclusion

In conclusion, the reproducibility of the two immobilization systems was compared and the CTV-PTV margins of the two hospitals were investigated. Results have indicated that there was a significant difference between the reproducibility of the two immobilization systems. Generally, alpha cradle performed better than the Hipfix system. This may be mainly due to the physical design of the two immobilization systems, although extraneous variables such as patient mobility could affect the results. More specifically, the CC direction contributed most to differences in reproducibility. While AP axis showed similar results, the Hipfix system performed slightly better than the alpha cradle system in ML direction, though it did not reach statistical significance. The CC direction for Hipfix system was also responsible for both the highest incidence of large and serious set-up errors due to the tendency of rotation of hips. The margins accounting for set-up errors were calculated. After including the suggested dimensions for prostate motion, they could then be more reliable when compared with the hospital protocols. It has been suggested that the margin for Hipfix in CC axis might not be adequate. Therefore, the margins may need to be modified as calculations related to other organ motions and target delineations were not calculated in this study.

Further prospective investigations with the same treatment protocols and more sophisticated set-up procedures as well as verification techniques are needed to determine whether Hipfix or alpha cradle systems perform better in terms of reproducibility and authorization of further margin reduction.

## Abbreviations

AP: Anterior-posterior; CC: Cranial-caudal; CT: Computed tomography; CTP: Conventional treatment position; CTV: Clinical target volume; DRRs: Digitally reconstructed radiographs; EBRT: External beam radiotherapy; GTV: Gross tumour volume; HL: Horizontal level; IMAT: Intensity-modulated arc therapy; IMRT: Intensity-modulated radiotherapy; IMS: Immobilization system; ML: Medial-lateral; ML: Mid-line; MLC: Multi-leaf collimator; OARs: Organs-at-risk; OBI: On-board imager; PTV: Planning target volume; PP: Principal plane; PSA: Prostate-specific antigen; PTV: Planning target volume; RA: RapidArc; SD: Standard deviation; SPSS: Statistical package for social sciences; SV: Seminal vesicle; TVE: Total vector error; VMAT: Volumetric-modulated arc therapy.

## Competing interests

The authors declare that they have no competing interests.

## Authors’ contributions

PW contributed to the conception, design, coordination and supervision of the study. KY interpreted the data, drafted and revised the manuscript. CS participated in the design of study and carried out data collection. WC participated in data acquisition and calculated total vector errors. HM interpreted the data and was responsible for the figures. SC performed the statistical analysis and helped to draft the manuscript. All authors carried out literature review, read and approved the final manuscript.

## Authors’ information

PW: Ph.D; LL.M (Wales); Assistant professor

KY: BSc. (Hons); Radiation Therapy graduate

CS: BSc. (Hons); Radiation Therapy graduate

WC: BSc. (Hons); Radiation Therapy graduate

HM: BSc. (Hons); Radiation Therapy graduate

SC: BSc. (Hons); Radiation Therapy graduate.
